# Multi-Physics Fields Based Nonlinear Dynamic Behavior Analysis of Air Bearing Motorized Spindle

**DOI:** 10.3390/mi11080723

**Published:** 2020-07-25

**Authors:** Guoda Chen, Yijie Chen, Qi Lu, Quanhui Wu, Minghuan Wang

**Affiliations:** 1 College of Mechanical Engineering, Zhejiang University of Technology, Hangzhou 310023, China; chenyj@zjut.edu.cn (Y.C.); luqi@zjut.edu.cn (Q.L.); wuqh@zjut.edu.cn (Q.W.); wangmh@zjut.edu.cn (M.W.); 2 Key Laboratory of Special Purpose Equipment and Advanced Processing Technology, Ministry of Education and Zhejiang Province, Zhejiang University of Technology, Hangzhou 310023, China; 3 State Key Laboratory of Fluid Power and Mechatronic Systems, Zhejiang University, Hangzhou 310027, China

**Keywords:** air journal bearing, unbalanced magnetic force, stability boundary, motorized spindle, rotor dynamic, Reynolds equation

## Abstract

The air bearing motorized spindle (ABMS) is the key component of the ultra-precision machine tool, which plays an important role in the ultra-precision machining process and directly influences machining accuracy. The influence of unbalanced magnetic force (UMF) on the nonlinear dynamic behavior of the ABMS is not understood clearly. To reveal the potential influence of the UMF, a mathematical model of the ABMS considering multiphysics fields is established. The variation trend of the UMF is simulated, and the nonlinear dynamic behavior of the ABMS is analyzed which emphasizes on the stability of the rotating shaft. It is shown that the UMF varies linearly at large rotor eccentricity which meets well with previous research, but it is noteworthy the UMF varies nearly to a quadratic function at small rotor eccentricity. The result of rotor dynamics shows that the UMF can change the converge position of the rotor center and the converge speed. Moreover, when at certain rotor mass and external load, the UMF can enlarge the stability boundary of the rotor. This research provides an example of analyzing the nonlinear dynamic behavior of the ABMS considering multiphysics fields which may help to the further investigation.

## 1. Introduction

The air bearing motorized spindle is commonly equipped in the ultra-precision machine, in which the rotor shaft is directly driven by usually a permanent magnet synchronous motor (PMSM) and supported by the air journal bearing [1,2,3,4]. Due to its compact structure, high efficiency, pollution free, high precision and other advantages, extensive studies of the ABMS have been conducted in the past few decades, which mainly focus on the dynamic characteristic of air journal bearing [5,6,7,8].

Dynamic coefficients method and restoring force method are two mainly analytical methods investigating the nonlinear dynamic behavior of bearing-rotor system [9,10]. Perturbation method is used the most which adapts the linearized form of parameters, the dynamic coefficients (i.e., stiffness and damping) can be obtained by solving the perturbation Reynolds equation [11,12,13]. Other method such as rational function approximation to the resulting impedances [14] and recursive least square method [15] are also used to obtain the dynamic coefficients of air bearing. When investigating the nonlinear dynamic behavior of the ABMS by applying the dynamic coefficients method, the dynamic coefficients are usually regarded as constant which improves the efficiency indeed, but ignore the influence caused by the variation of the dynamic coefficients [16,17,18]. The dynamic coefficients method is based on the acquisition of dynamic coefficients while the restoring force method can be obtained directly by solving the transient Reynolds equation. When considering the time-varying dynamic coefficients, massive extra work needs to be done then. By contrast, restoring force appears to be more efficient [19]. The transient Reynolds equation can be solved using finite element method (FEM) or finite difference method (FDM), due to less complex meshing and iterative, the FDM gets more preference which is easy to obtain the transient restoring force, especially when considering multiphysics fields [20,21,22]. The nonlinear dynamic behavior of air bearing spindle system has been investigated under different rotor mass, bearing number, non-stationary loading force and unbalanced mass [23,24,25,26]. To the ABMS, however, the influence of Electromagnetic factors on the dynamic behavior of the ABMS system is not understood clearly. It had been pointed out the existence of stability boundary of air journal bearing, and the stability boundary varying with rotating speed is also investigated [27,28]. To the ABMS, however, the influence of Electromagnetic factors to the stability of the system may not be ignored. It has been investigated the existence of the UMF which is the manifestation of electromagnetic effect when the rotor eccentricity happens [29,30]. Xu et al. [31] investigated the influence of the UMF to the dynamic behavior of motor rotor, the limit cycle with and without unbalanced mass was analyzed. Wu et al. [32,33] investigated the UMF in the ABMS and its effect on surface generation in turning which stressed the underlying significance of electromagnetic factors of the ABMS. From the previous research, the influence of electromagnetic factors on the dynamic behavior of the ABMS remains to be further explored, especially when considering multiphysics fields.

Therefore, the nonlinear dynamic behavior analysis of air bearing motorized spindle considering multiphysics fields is investigated in this study. The FDM is adopted to discrete the transient Reynolds equation, and the restoring force is obtained by solving the transient Reynolds equation. The UMF is calculated in a 2D PMSM model with eccentricity using COMSOL Multiphysics software. The restoring force and the UMF are coupled within the dynamic model in which the rotor is treated as a rigid rotor.

## 2. Mathematical Modeling

### 2.1. Modeling for Restoring Force of Air Journal Bearing

The air journal bearings model incorporates the following assumptions: the flow is isothermal, the gas viscosity is assumed to be constant, the mass flow loss caused by the end leakage of the bearing is neglected, there is no velocity slip at the boundary, the air is an ideal gas.

The transient pressure distribution in the clearance between the air journal bearing and the rotor shaft is modeled by the transient Reynolds equation as follows:

(1) $$\frac{\partial }{\partial x}\left(\frac{ph^{3}}{\mu }\frac{\partial p}{\partial x}\right)+\frac{\partial }{\partial z}\left(\frac{ph^{3}}{\mu }\frac{\partial p}{\partial z}\right)=12\frac{\partial }{\partial t}\left(ph\right)+6\omega \frac{\partial }{\partial x}\left(ph\right)$$

where x is the circumferential coordinates of air journal bearing, z is the axial coordinate of air journal bearing, h is the air film thickness of air film, p is the pressure of air film, μ is the dynamic viscosity of air, ω is the rotating angular speed of the rotor, t represents the time.

The air film thickness is given by:

(2) $$h=C_{r}\left(1+\varepsilon \cos\left(\theta -\theta_{a}\right)\right)$$

where $C_{r}$ is the radial clearance air journal bearing, ε is the eccentricity of the rotor, θ is the circumferential angle of air journal bearing, $\theta_{a}$ is the eccentric angle of the rotor.

The eccentricity of the rotor and the eccentric angle of the rotor are given as follows:

(3) $$\varepsilon =\frac{1}{C_{r}}\sqrt{{\left(e_{x}\right)}^{2}+{\left(e_{y}\right)}^{2}}$$

(4) $$\theta_{a}=\{\begin{array}{c} \mathrm{arctg}\frac{e_{y}}{e_{x}}\;\left(e_{x}\geq 0\right)\\ \mathrm{arctg}\frac{e_{y}}{e_{x}}+\pi \;(e_{x}<0) \end{array}$$

where $e_{x}$ is the eccentricity of the rotor in x direction, $e_{y}$ is the eccentricity of the rotor in y direction.

The dimensionless form of the transient Reynolds equation is given by:

(5) $$\frac{\partial }{\partial \theta }\left(H^{3}\frac{\partial P^{2}}{\partial \theta }\right)+\frac{\partial }{\partial Z}\left(H^{3}\frac{\partial P^{2}}{\partial Z}\right)=2\Lambda \frac{\partial }{\partial \tau }\left(PH\right)+\Lambda \frac{\partial }{\partial \theta }\left(PH\right)$$

where Λ is the bearing number.

The dimensionless parameters are defined as follows:

$$P=\frac{p}{P_{r}},\;H=\frac{h}{C_{r}},\;\theta =\frac{x}{R},\;Z=\frac{z}{R},\;\tau =\omega t,\;\Lambda =\left(\frac{12\mu \omega }{P_{r}}\right){\left(\frac{R}{C_{r}}\right)}^{2}$$

where R is the radius of air journal bearing, $P_{r}$ is the preference pressure. The air journal bearing is an aerodynamic bearing when there is no external air supplement.

**Figure 1:**
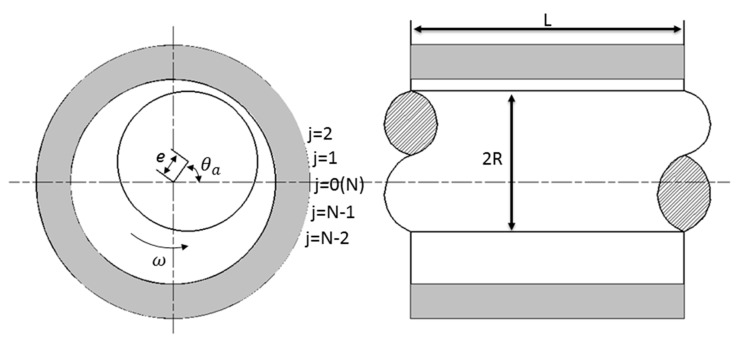
Schematic structure of the air journal bearing.

**Figure 2:**
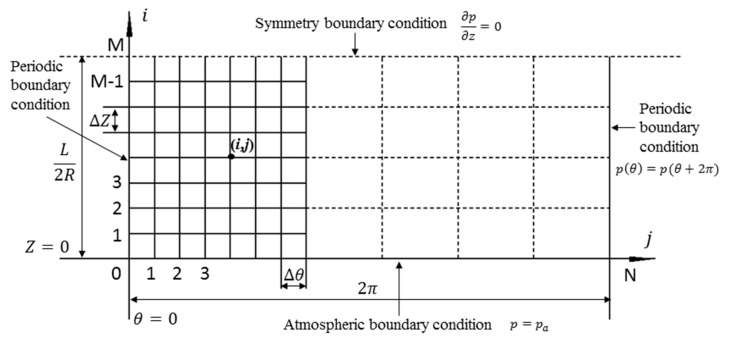
Computational domain of air journal bearing.

Figure 1 shows the structure schematic of the air journal bearing [26]. The structure of air journal bearing is symmetrical, thus only half of the computational domain is needed which can be realized by setting the symmetry boundary condition, as shown in Figure 2, the periodic boundary condition and atmosphere boundary condition are also defined. The computational domain is θ(0:N+1) and Z(0:M).

Submitting the expression $S=P^{2}$, the central difference method is employed to discrete the variable in the θ and Z directions. In the absence of any other treatment, the dimensionless transient Reynolds equation is an implicit equation contains five unknown variables within every time step which is hard to be solved, thus the ADI method [10] is used to simplify the implicit equation. According to the rules of ADI method, the increment in the marching direction θ and Z simultaneously between the time steps of n and n+1 are reorganized.

At the beginning of the computation, the pressure distribution is assumed to be known at the time step n. At the time steps of n+1/2, only the increment in the marching direction Z is carried out, and when it goes to the time steps of n+1, the increment in the marching direction θ is carried out. The discretized dimensionless transient Reynolds equation employed ADI method is as follows:

(6) $$A_{i,j}S^{n+\frac{1}{2}}{}_{i-1,j}+B_{i,j}S^{n+\frac{1}{2}}{}_{i,j}+C_{i,j}S^{n+\frac{1}{2}}{}_{i+1,j}=D_{i,j}$$

(7) $$A_{i,j}=-\frac{H^{n}{{}_{i,j}}^{3}}{{\left(∆Z\right)}^{2}}+3H^{n}{{}_{i,j}}^{2}\frac{H^{n}{}_{i+1,j}-H^{n}{}_{i-1,j}}{{\left(2∆Z\right)}^{2}}$$

(8) $$B_{i,j}=\frac{2H^{n}{{}_{i,j}}^{3}}{{\left(∆Z\right)}^{2}}+\frac{\Lambda H^{n}{}_{i,j}}{P^{n}{}_{i,j}∆\tau }$$

(9) $$C_{i,j}=-\frac{H^{n}{{}_{i,j}}^{3}}{{\left(∆Z\right)}^{2}}-3H^{n}{{}_{i,j}}^{2}\frac{H^{n}{}_{i+1,j}-H^{n}{}_{i-1,j}}{{\left(2∆Z\right)}^{2}}$$

(10) $$\begin{array}{c} D_{i,j}=3H^{n}{{}_{i,j}}^{2}\frac{H^{n}{}_{i,j+1}-H^{n}{}_{i,j-1}}{2∆\theta }\frac{S^{n}{}_{i,j+1}-S^{n}{}_{i,j-1}}{2∆\theta }\\ +H^{n}{{}_{i,j}}^{3}\frac{S^{n}{}_{i,j+1}-2S^{n}{}_{i,j}+S^{n}{}_{i,j-1}}{{\left(∆\theta \right)}^{2}}-\frac{\Lambda H^{n}{}_{i,j}}{2P^{n}{}_{i,j}}\frac{S^{n}{}_{i,j+1}-S^{n}{}_{i,j-1}}{2∆\theta }-\Lambda P^{n}{}_{i,j}\frac{H^{n}{}_{i,j+1}-H^{n}{}_{i,j-1}}{2∆\theta }\\ \begin{array}{c} -\Lambda P^{n}{}_{i,j}\frac{H^{n}{}_{i,j+1}-H^{n}{}_{i,j-1}}{2∆\theta }+\frac{\Lambda H^{n}{}_{i,j}S^{n}{}_{i,j}}{P^{n}{}_{i,j}∆\tau }-2\Lambda P^{n}{}_{i,j}\frac{H^{n+1/2}{}_{i,j}-H^{n}{}_{i,j}}{∆\tau } \end{array} \end{array}$$

where $S^{n+\frac{1}{2}}{}_{i-1,j}$, $S^{n+\frac{1}{2}}{}_{i,j}$, $S^{n+\frac{1}{2}}{}_{i+1,j}$, are the unknown parameters at the time step of n+1/2, $H^{n+1/2}{}_{i,j}$ can be acquired by calculating the dynamic equation and the Equation (6) can be solved by the Thomas method,

(11) $$E_{i,j}S^{n+1}{}_{i,j-1}+F_{i,j}S^{n+1}{}_{i,j}+G_{i,j}S^{n+1}{}_{i,j+1}=I_{i,j}$$

(12) $$E_{i,j}=-\frac{H^{n+1/2}{{}_{i,j}}^{3}}{{\left(∆\theta \right)}^{2}}-\frac{\Lambda H^{n+1/2}{}_{i,j}}{4P^{n+1/2}{}_{i,j}∆\theta }+3H^{n+1/2}{{}_{i,j}}^{2}\frac{H^{n+1/2}{}_{i,j+1}-H^{n+1/2}{}_{i,j-1}}{{\left(2∆\theta \right)}^{2}}$$

(13) $$F_{i,j}=\frac{2H^{n+1/2}{{}_{i,j}}^{3}}{{\left(∆\theta \right)}^{2}}+\frac{\Lambda H^{n+1/2}{}_{i,j}}{P^{n+1/2}{}_{i,j}∆\tau }$$

(14) $$G_{i,j}=-\frac{H^{n+1/2}{{}_{i,j}}^{3}}{{\left(∆\theta \right)}^{2}}+\frac{\Lambda H^{n+1/2}{}_{i,j}}{4P^{n+1/2}{}_{i,j}∆\theta }-3H^{n+1/2}{{}_{i,j}}^{2}\frac{H^{n+1/2}{}_{i,j+1}-H^{n+1/2}{}_{i,j-1}}{{\left(2∆\theta \right)}^{2}}$$

(15) $$\begin{array}{c} I_{i,j}=3H^{n+1/2}{{}_{i,j}}^{2}\frac{H^{n+1/2}{}_{i+1,j}-H^{n+1/2}{}_{i+1,j}}{2∆Z}\frac{f^{n+1/2}{}_{i+1,j}-f^{n+1/2}{}_{i-1,j}}{2∆Z}\\ +H^{n+1/2}{{}_{i,j}}^{3}\frac{S^{n+1/2}{}_{i+1,j}-2S^{n+1/2}{}_{i,j}+S^{n+1/2}{}_{i-1,j}}{{\left(∆Z\right)}^{2}}-\Lambda P^{n+1/2}{}_{i,j}\frac{H^{n+1/2}{}_{i,j+1}-H^{n+1/2}{}_{i,j-1}}{2∆\theta }\\ \begin{array}{c} +\frac{\Lambda H^{n+1/2}{}_{i,j}S^{n+1/2}{}_{i,j}}{P^{n+1/2}{}_{i,j}∆\tau }-2\Lambda P^{n+1/2}{}_{i,j}\frac{H^{n+1}{}_{i,j}-H^{n+1/2}{}_{i,j}}{∆\tau } \end{array} \end{array}$$

where $S^{n+1}{}_{i,j-1}$, $S^{n+1}{}_{i,j}$, $S^{n+1}{}_{i,j+1}$, are the unknown parameters at the time step of n+1/2, $H^{n+1}{}_{i,j}$ can be acquired by calculating the dynamic equation, and the Equation (11) can be solved by the Thomas method.

The ADI method is not unconditionally stable for solving the nonlinear equations, thus the step time ∆τ should be less than 0.01. By solving the transient Reynolds equation, the transient air pressure is acquired, and the restoring force of single air journal bearing can be obtained as follows:

(16) $$f_{bx}=P_{r}R^{2}\int_{0}^{\frac{L}{R}}\int_{0}^{2\pi }P\left(Z,\theta ,\tau \right)\;\cos\theta \;d\theta \;\mathrm{dZ}$$

(17) $$f_{by}=P_{r}R^{2}\int_{0}^{\frac{L}{R}}\int_{0}^{2\pi }P\left(Z,\theta ,\tau \right)\;\sin\theta \;d\theta \;\mathrm{dZ}$$

### 2.2. Modeling for UMF of PMSM

The UMF is mainly resulted from the asymmetrical distribution of magnetic flux density due to the rotor eccentricity, which means ideally, the UMF is nearly to zero in a symmetric PMSM with no rotor eccentricity. According to the Maxwell stress tensor method, the 2D magnetic forces in the radial and tangential direction can be expressed as follows [34]:

(18) $$f_{r}=\int_{0}^{2\pi }\frac{1}{2\mu_{0}}\left(B_{r}^{2}-B_{\theta }^{2}\right)\;r\;d\theta$$

(19) $$f_{\theta }=\int_{0}^{2\pi }\frac{B_{r}B_{\theta }}{\mu_{0}}\;r\;d\theta$$

where $B_{r}$ is the radial flux density, $B_{\theta }$ is the tangential flux density, $\mu_{0}$ is the permeability of the air.

**Figure 3:**
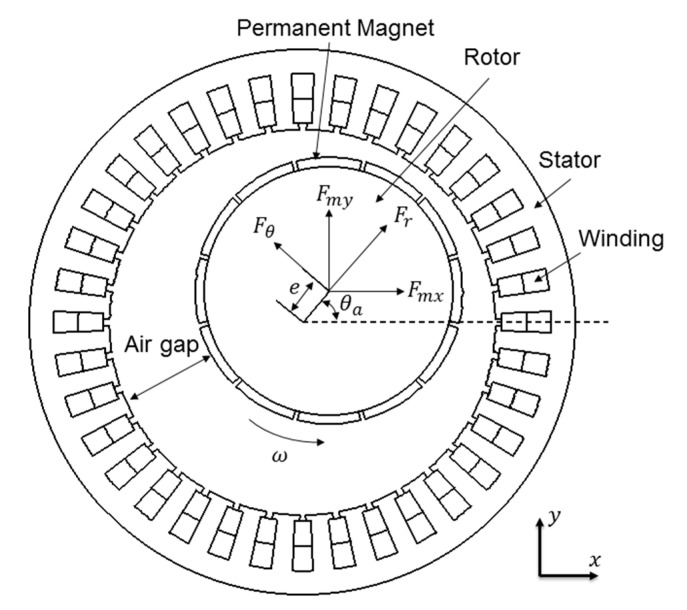
Schematic structure of the motor.

Figure 3 shows the schematic structure of motor with eccentric rotor, and the magnetic force in cartesian coordinates can be expressed as:

(20) $$f_{mx}=f_{r}\cos\theta_{a}+f_{\theta }\sin\theta_{a}$$

(21) $$f_{my}=f_{r}\sin\theta_{a}+f_{\theta }\cos\theta_{a}$$

### 2.3. Modeling for Rotor Dynamic System

The rotor here is regarded as a rigid rotor, and the tilting of the rotor is neglected. The numeric model is employed to obtain the rotor center trajectory based on multiphysics fields coupling. Moreover, the dimensionless acceleration, velocity and position of the rotor center can be calculated by:

(22) $$A_{x}=\frac{d^{2}X}{d\tau^{2}}=\frac{F_{ex}+F_{mx}-F_{bx}}{M}$$

(23) $$A_{y}=\frac{d^{2}Y}{d\tau^{2}}=\frac{F_{ey}+F_{mx}-F_{by}}{M}$$

(24) $$V_{x}=V_{x0}+A_{x}∆\tau$$

(25) $$V_{y}=V_{y0}+A_{y}∆\tau$$

(26) $$X=X_{0}+V_{x}∆\tau +\frac{1}{2}A_{x}∆\tau^{2}$$

(27) $$Y=Y_{0}+V_{y}∆\tau +\frac{1}{2}A_{y}∆\tau^{2}$$

where $f_{ex}$, $f_{ey}$ are the dimensionless external force, $f_{mx}$, $f_{my}$ are the dimensionless magnetic force, $f_{bx}$, $f_{by}$ are the dimensionless bearing restoring force, $V_{x0},$ $V_{y0}$ are the velocity of the rotor center at the last time step, $X_{0}$, $Y_{0}$ are the position of the rotor center at the last time step,

M is the dimensionless mass of the rotor. Moreover, the dimensionless parameters are defined as follows:

$$F_{ex}=\frac{f_{ex}}{P_{r}R^{2}},\;F_{ey}=\frac{f_{ey}}{P_{r}R^{2}},\;F_{mx}=\frac{f_{mx}}{P_{r}R^{2}},\;F_{ey}=\frac{f_{my}}{P_{r}R^{2}},\;F_{bx}=\frac{f_{bx}}{P_{r}R^{2}},\;F_{ey}=\frac{f_{by}}{P_{r}R^{2}},\;X=\frac{x}{C_{r}},\;Y=\frac{y}{C_{r}},\;M=\frac{mC_{r}\omega^{2}}{P_{r}R^{2}}$$

**Figure 4:**
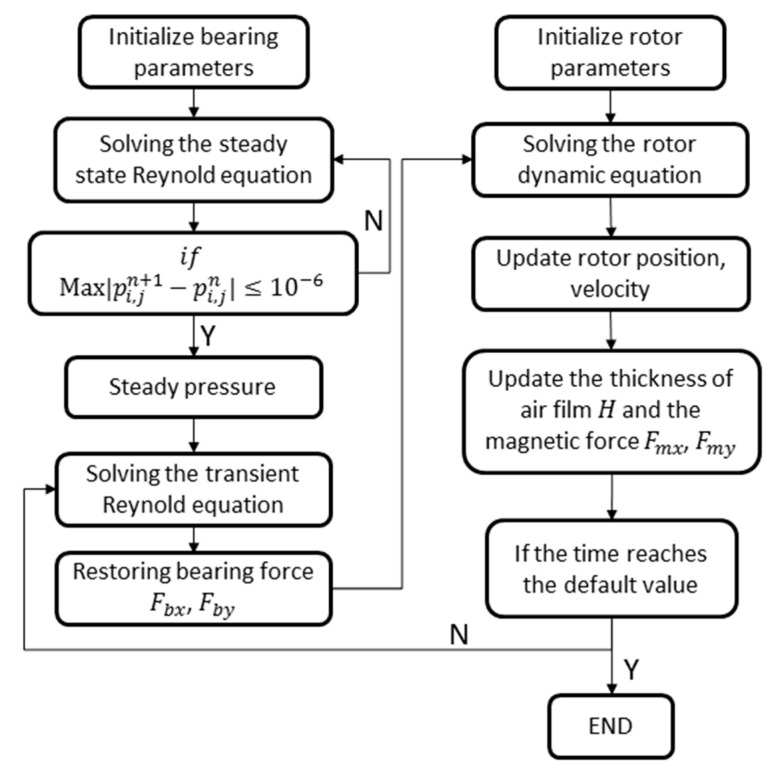
The flow chat for calculating the coupled dynamic equation.

The air journal bearing is at rest in the initial condition, thus $V_{x0}=V_{y0}=0$ at the initial time. The flow chat for calculating the dynamic equation of the rotor system considering multiphysics fields is shown in Figure 4.

## 3. Numeric Simulation of the Mathematical Model

### 3.1. Simulation of the PMSM Model

The PMSM is modeled based on FEM in COMSOL Multiphysics software. Table 1 lists the detailed parameters of the motor. Moreover, the boundary condition of the simulation is listed as follows: The stator outer circle is selected as the boundary which has no magnetic leakage. The residual magnetic flux density of permanent magnets is set to 1 T, and the magnetic poles alternates on the circumference. The rotor speed is set as the form of rotating angle (ω/60∙time), the three-phase alternating currents are (A: 6·cos(2·π·ω/60·time), B: 6·cos(2·π·ω/60·time−2·π/3), C: 6·cos(2·π·ω/60·time−2·π/3)) input the winding. The magnetic scalar potential and magnetic vector potential are set to zero at initial time

**Figure 5:**
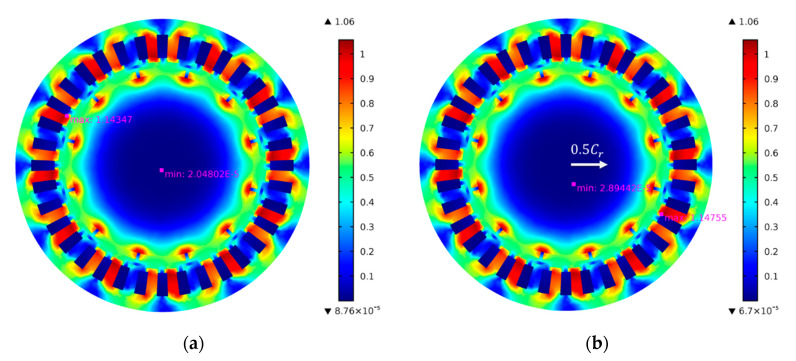
Flux density of the motor at 0.02 s with different eccentricities. (**a**) 0% eccentricity; (**b**) 50% eccentricity.

Figure 5a,b shows the flux density of the motor at 0.02 s with 0% eccentricity (It should be noted that the rotor eccentricity represents the eccentricity at the air journal bearing rather than the motor) and 50% eccentricity, respectively, it can be seen that the both the minimum and the maximum flux density of the motor increased when the rotor is eccentric. By integrating the flux density along the surface of the rotor, the UMF can be obtained. Wang et al. [29] pointed out that the UMF increases almost linearly with the eccentricity which was also proved by Wu et al. [32]. However, the rotor eccentricity in their research is at the motor which is much larger than the eccentricity at air journal bearing, it means the variation of the UMF may not vary linearly at small eccentricity.

**Figure 6:**
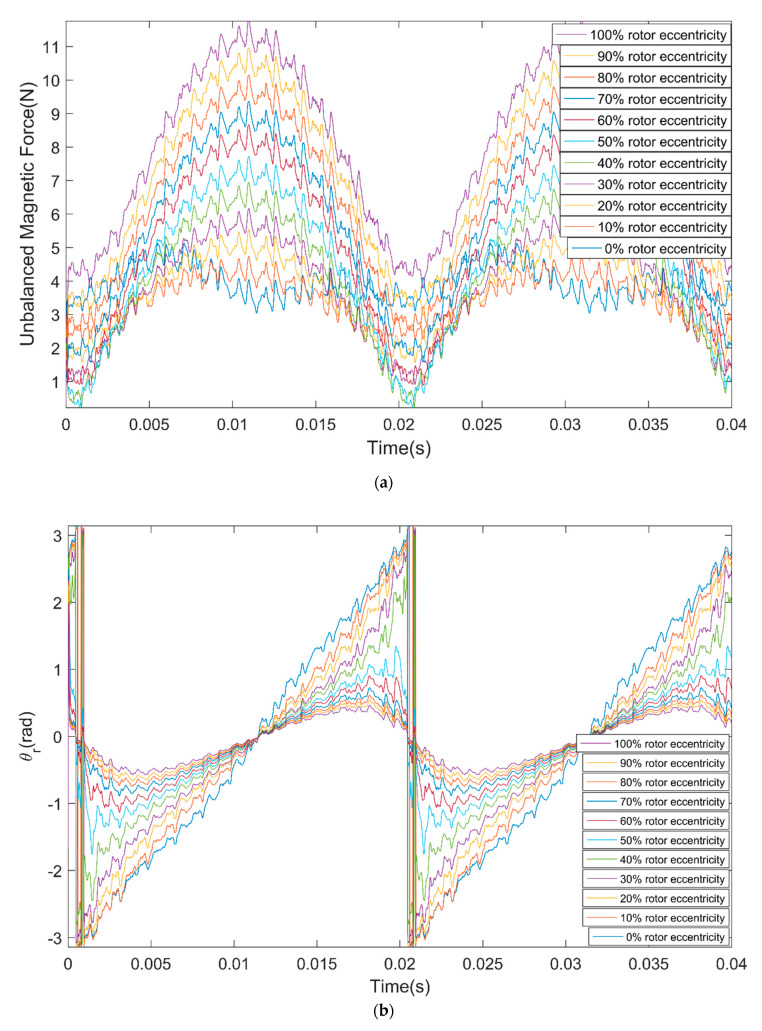
Variation of the unbalanced magnetic force (UMF) and its direction angle with time under different rotor eccentricity. (**a**) UMF; (**b**) direction angle of the UMF $\theta_{r}$.

**Figure 7:**
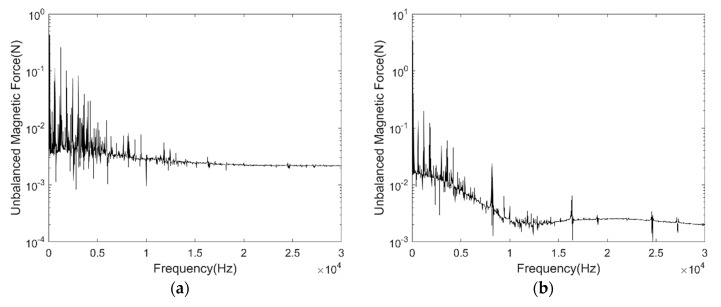
Frequency spectrum of the UMF. (**a**) 0% eccentricity; (**b**) 100% eccentricity.

Figure 6a shows the UMF under different rotor eccentricity vary with time, Figure 6b shows the direction angle of the UMF $\theta_{r}$ under different rotor eccentricity vary with time. It can be seen that the UMF changes periodically, the amplitude of the UMF increases with the eccentricity. The direction angle of the UMF should be the eccentric direction ideally, but it shows notable periodic fluctuation with time when at small eccentricity. Figure 7a,b shows the frequency spectrum of the UMF under 0% eccentricity and 100% eccentricity, respectively. The amplitude of the low frequency component increases significantly.

**Figure 8:**
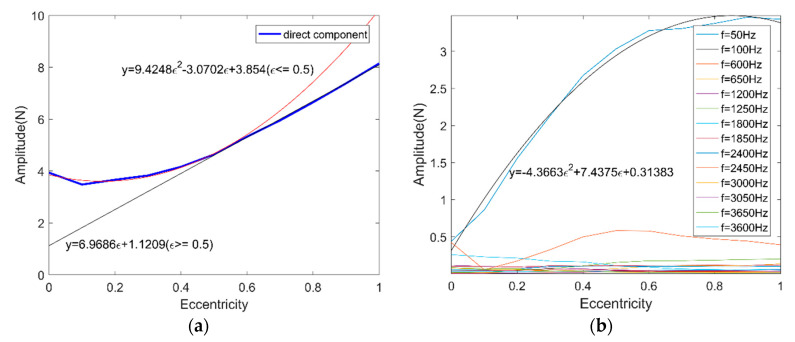
Frequency component variation of the spectrum of the UMF. (**a**) Direct component; (**b**) other frequency components.

Figure 8a shows the direct component variation and its fitted curve of the spectrum of the UMF under different eccentricity. When the rotor eccentricity is relatively large, the UMF increases linearly with eccentricity which is consistent with previous research, but when the eccentricity is small (less than 50%, approximately), the UMF is close to a quadratic function with eccentricity. Figure 8b depicted the main frequency component variation of the spectrum of the UMF under different eccentricity. The frequency component f = 50 Hz leads the dominate position with the highest amplitude, thus only f = 50 Hz is considered as the main contributor to the fluctuation of the UMF.

**Figure 9:**
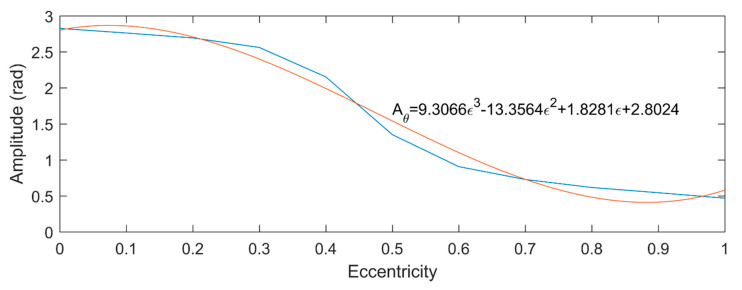
Amplitude fluctuation of $\theta_{r}$.

**Figure 10:**
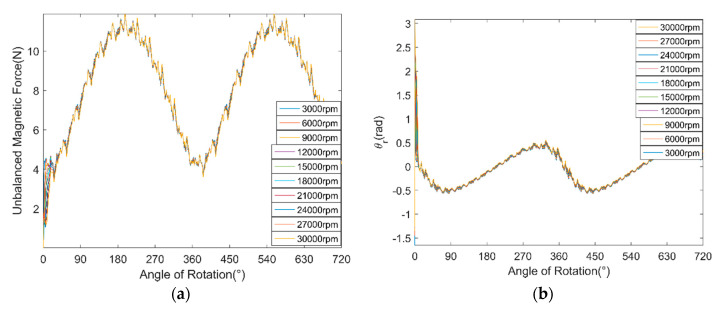
Variation of the UMF and its direction angle at different rotor speed. (**a**) UMF; (**b**) direction angle of the UMF.

Figure 9 shows the amplitude fluctuation of $\theta_{r}$, and its varying trend is also fitted by a cubic curve. The fluctuation of $\theta_{r}$ is regarded as sinusoidal function. Figure 10a,b shows the UMF and its direction angle at different rotor speed, respectively. It can be seen that the rotation speed of the rotor hardly influences the magnitude of the UMF and its direction angle, but only change the frequency of them.

The variation function of the UMF and its direction angle were obtained based on the simulation of the PMSM model. To simplify the following simulation of nonlinear dynamic behavior analysis of the ABMS, only one decimal is reserved. The variation function of the UMF and its direction angle can be expressed as:

$$\begin{array}{c} f_{mx}=\{\begin{array}{c} {}[\left(-4.4\varepsilon^{2}+7.4\varepsilon +0.3\right)\sin\left(2\pi ∆\frac{\omega }{60}t\right)+\left(9.4\varepsilon^{2}-3.1\varepsilon +3.8\right)]∆sin\theta_{r}\;\left(\varepsilon \leq 0.5\right)\\ \;\left[\left(-4.4\varepsilon^{2}+7.4\varepsilon +0.3\right)\sin\left(2\pi ∆\frac{\omega }{60}t\right)+\left(7.0\varepsilon +1.1\right)\right]∆sin\theta_{r}\;\left(\varepsilon \geq 0.5\right) \end{array}\\ f_{my}=\{\begin{array}{c} {}[\left(-4.4\varepsilon^{2}+7.4\varepsilon +0.3\right)\sin\left(2\pi ∆\frac{\omega }{60}t\right)+\left(9.4\varepsilon^{2}-3.1\varepsilon +3.8\right)]∆cos\theta_{r}\;\left(\varepsilon \leq 0.5\right)\\ \;\left[\left(-4.4\varepsilon^{2}+7.4\varepsilon +0.3\right)\sin\left(2\pi ∆\frac{\omega }{60}t\right)+\left(7.0\varepsilon +1.1\right)\right]∆cos\theta_{r}\;\left(\varepsilon \geq 0.5\right) \end{array}\\ \theta_{r}=\left(9.3\varepsilon^{3}-13.4\varepsilon^{2}+1.8\varepsilon +2.8\right)\sin\left(2\pi ∆\frac{\omega }{60}t\right) \end{array}$$

### 3.2. Simulation of Nonlinear Dynamic Behavior of the ABMS

In this section, the multifield coupling model of rotor dynamics is analyzed. The detailed data of the air journal bearing is listed as follows:

$$L=0.1\;m,\;R=0.05\;m,\;C_{r}=2\times 10^{-5}m,\;\mu =1.82\times 10^{-5}\;P_{a}∆s,\;P_{r}=4\;\mathrm{atm}.$$

Set the rotor to a certain initial position, chose the parameter ω=3000 rpm (Λ=1.058), a static load $f_{e}=100-N$ was loaded on the rotor ($F_{e}=0.0987$). Moreover, the dynamic behavior of the rotor with and without considering the UMF was investigated.

**Figure 11:**
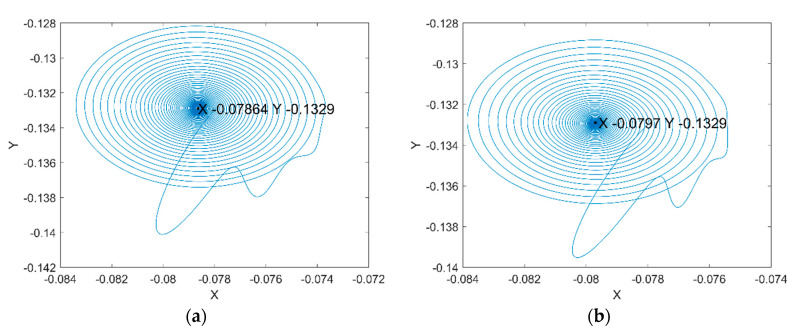
Rotor center trajectory at $F_{e}=0.0987,\;M=0.1071$. (**a**) Without considering the UMF; (**b**) with considering the UMF.

**Figure 12:**
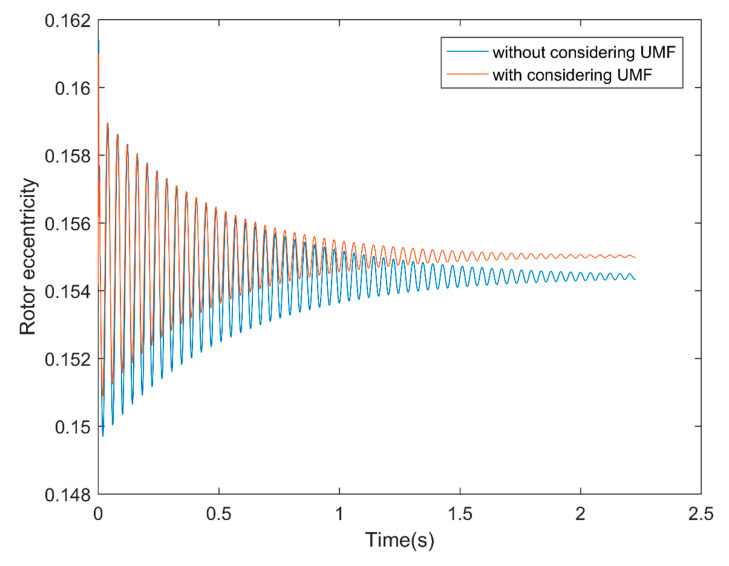
Rotor eccentricity variation with and without considering the UMF.

Figure 11a shows the rotor center trajectory without considering the UMF at $F_{e}=0.0987$, M=0.1071 and Figure 11b shows the rotor center trajectory with considering the UMF at the same condition. It can be seen that the UMF changes the converge position, but not obvious. In the meanwhile, the UMF helps to accelerate the convergence of the rotor center trajectory as shown in Figure 12.

**Figure 13:**
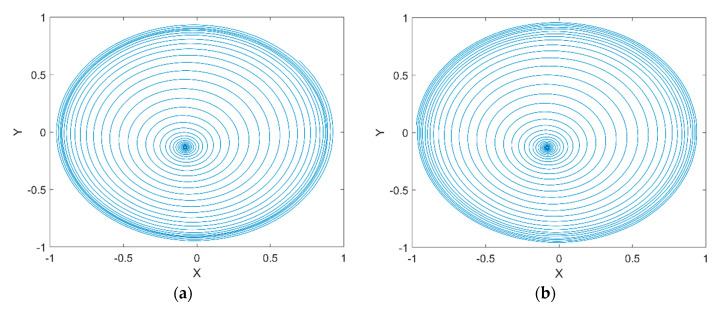
Rotor center trajectory at $F_{e}=0.0987$, M=0.2825 (**a**) without considering the UMF; (**b**) with considering the UMF.

**Figure 14:**
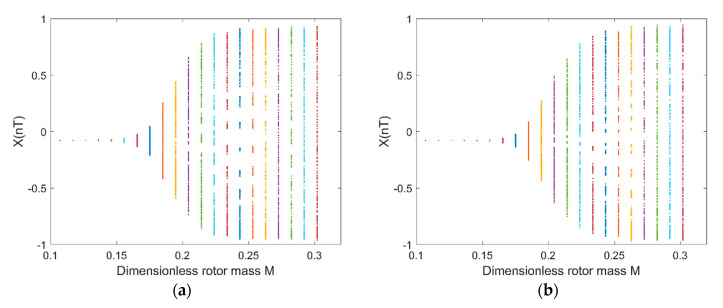
Bifurcation diagram: displacement in X-direction with dimensionless rotor mass M. (**a**) Without considering the UMF; (**b**) with considering the UMF.

Figure 13a shows the rotor center trajectory without considering the UMF at $F_{e}=0.0987$, M=0.2825 and Figure 13b shows the rotor center trajectory with considering the UMF at the same condition. The rotor center trajectory diverges at bigger rotor mass which means there is a threshold of rotor mass that determines whether the rotor trajectory is convergent or divergent. Figure 14a shows the bifurcation diagram of displacement of rotor center in X direction with dimensionless rotor mass without considering the UMF; Figure 14b shows the bifurcation diagram with considering the UMF. The result shows the threshold of dimensionless rotor mass is less than 0.15 without considering the UMF, while the threshold of dimensionless rotor mass exceeds 0.15 with considering the UMF, that is, the UMF changes the stability threshold of the rotor mass when at a certain static external load.

**Figure 15:**
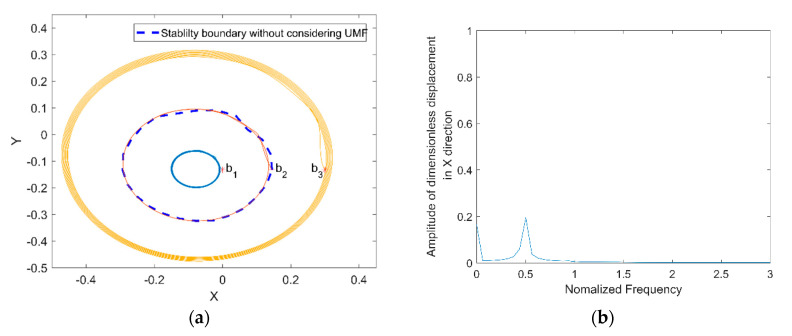
Rotor center trajectory without considering the UMF at $F_{e}=0.0987,\;M=0.1403.$ (**a**) Rotor center trajectory from different initial positions; (**b**) power spectrum of displacement of the rotor center trajectory starts from $b_{2}$.

Yang et al. [19] pointed out that the stability of the rotor depends on the initial position when at certain rotor mass and external load; it is also investigated by Khonsari et al. [27]. Therefore, the stability of the rotor from different initial position is investigated and compared with and without considering the UMF. Figure 15a shows the rotor trajectory from different initial position without considering the UMF at $F_{e}=0.0987$, M=0.1403. The coordinates of three initial positions are $b_{1}$(0, −0.133), $b_{2}$(0.145, −0.133) and $b_{3}$(0.3, −0.133). The blue dashed line is the stability boundary acquired by Khonsari’s method. When the initial position is within the stability boundary such as position $b_{1}$, the rotor center trajectory converges to a certain position; when the initial position is on the stability boundary such as position $b_{2}$, the rotor center trajectory is an unstable limited circle; when the initial position is out of the stability boundary such as $b_{3}$, the rotor center trajectory diverges outward to be unstable. The power spectrum of displacement of the rotor center trajectory start from $b_{2}$ is shown in Figure 15b, it shows that the whirl frequency of the rotor is about half of the rotating frequency of the rotor, that is the famous “half speed whirl” which prove the correctness of the calculation.

**Figure 16:**
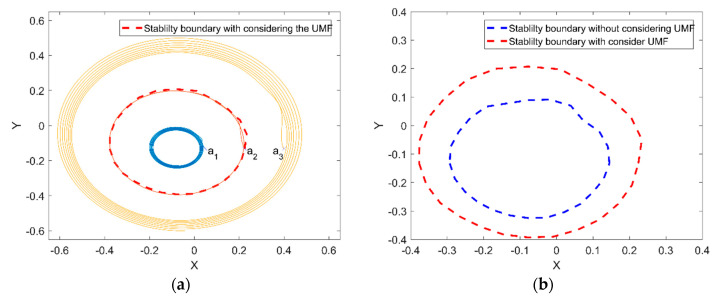
Rotor center trajectory with considering the UMF at $F_{e}=0.0987,\;M=0.1403.\;$(**a**) Rotor center trajectory from different initial position; (**b**) comparison of stability boundary with and without considering the UMF.

Figure 16a shows the rotor center trajectory from different initial position with considering the UMF at $F_{e}=0.0987$, M=0.1403. The coordinates of three initial positions are $a_{1}$(0.05, −0.133), $a_{2}$(0.2245, −0.133) and $a_{3}$(0.4, −0.133). The red dashed line is the stability boundary with considering the UMF. The variation trend of the rotor center trajectory from different initial position (i.e., within the stability boundary, on the stability boundary and out of the stability boundary) is the same. However, is should be noticed the variation of the stability boundary after considering the UMF, the comparison of the stability boundary with and without considering the UMF is shown in Figure 16b. It can be seen that the UMF enlarges the stability boundary conspicuously.

## 4. Discussion

The investigation is based on the mathematical model, the result of the UMF meets well with previous research at large rotor eccentricity. However, the variation of the UMF at small rotor eccentricity showed nonlinear trend in the simulation. The fluctuation of the UMF is due to the fluctuation of the winding currents, though the PMSM model is symmetric, the UMF exists. The eccentricity of motor rotor results in the asymmetrical magnetic field at the air gap which contributes to the UMF and enlarge the amplitude of it. The probable explanation to the variation trend of the UMF is as follows: when at large eccentricity, the magnetic field at the air gap is severely imbalanced and its influence on the UMF far exceeds the effect of the winding currents, and the UMF varies linearly as the eccentricity; when at small eccentricity case, the influence of imbalanced magnetic field is not dominant, that is the influence of the winding currents emerges, thus the variation of the UMF at small rotor eccentricity showed nonlinear trend. Certainly, the result demands further experiment validation.

In the result of the nonlinear dynamic behavior analysis of the ABMS, it showed that the UMF changes the converge position but not obvious which is mainly because that the UMF is relatively small compared to the external load. The influence of the UMF on the convergence of the rotor is not fully understood, we guess it is due to the nonlinear property of the air film. When at larger eccentricity, the air film may have larger dynamic stiffness which helps to accelerate the convergence speed. The same phenomenon is also observed when apply larger external load. The result also showed that the rotor center trajectory depends on the initial position when at certain rotor mass and external load, Yang et al. [19] gave the approximate critical value of the rotor mass at a certain external load. We had done massive calculation and found that the critical value can be renovated by adapting higher precision. To our knowledge, the exact critical value which result in unconditional stable and unstable consequence can only be approached but not obtained exactly. Both the external force and the rotor mass have influence on the stability boundary, and the result shows that the stability boundary with considering the UMF exceeds the stability without considering the UMF, thus the possible explanation for it is that the UMF is potentially equivalent to the increase of the external force, it also needs to be further explored. This study has certainly limitations, only the journal bearing at the ideal case is investigated (i.e., the rotor is considered as a rigid rotor), the thrust bearing is not modeled and the flexibility of the rotor is neglected, thus, the tilt of the rotor is neglected which is result of the joint interaction of journal bearing, thrust bearing and the rotor. The model in this study is a multifield coupling model which demands massive calculation. Thus, only the dynamic characteristic of the ABMS at certain rotating speed and external load was analyzed, and these parameters should be analyzed in further research, as well as the influence of the inherent unbalanced rotor mass with considering the UMF. The research gives an example of analyzing the influence of the UMF on the ABMS which worth further investigation.

## 5. Conclusions

In this study, a mathematical model of the ABMS considering the UMF is established and the dynamic behavior of ABMS is analyzed. The following conclusions can be drawn:

1. When the PMSM is at small rotor eccentricity status, the unbalanced magnetic shows quadratic variation trend with rotor eccentricity while it changes linearly at large rotor eccentricity status;
2. The existence of the UMF can slightly change the final convergent position of the rotor center and it can accelerate the convergence of the rotor center to some extent;
3. The UMF can change the bifurcation threshold of rotor mass at a certain external load. The other form of its manifestation is that the UMF can enlarge the stability boundary at certain rotor mass and external load.

## Figures and Tables

**Table 1:** Part parameters of spindle motor.

Parameters (Unit)	Rotor	Stator
Outer stator diameter (mm)	150	100
Inner rotor diameter (mm)	106	–
Number of slots	36	
Number of poles	12	
Motor effective length (mm)	80	
Rated speed (r/min)	3000	
Air gap (mm)	2	
Winding form	Three phases of double layer winding	
